# Pain Assessment in Impaired Cognition (PAIC15) Instrument: Cutoffs Against Three Standards

**DOI:** 10.1093/geroni/igab046.630

**Published:** 2021-12-17

**Authors:** Jenny van der Steen, Margot de Waal, Wilco Achterberg

**Affiliations:** Leiden University Medical Center, Leiden, Zuid-Holland, Netherlands

## Abstract

Observational pain scales can help identify pain in persons with impaired cognition including dementia who may have difficulty expressing pain verbally. The Pain Assessment in Impaired Cognition-15 (PAIC15) observational pain scale covers 15 important items that are indicative of pain, but it is unclear how likely pain is for persons with each summed score (theoretical range 0-45). The goal of our study was to determine sensitivity and specificity of cut offs for probable pain on the PAIC15 against three possible standards. We determined cut offs against (1) self report when able, (2) the established Pain Assessment in Advanced Dementia (PAINAD) cut off of 2, and (3) observer’s overall estimate based on a series of systematic observations. We used data of 238 nursing home residents with dementia who were observed by their physician in training or nursing staff in the context of an evidence-based medicine (EBM) training study, with 137 residents assessed twice. The area under the ROC curve was excellent against the PAINAD cut off (□0.8) at both assessments, but acceptable or less than acceptable for the other two standards. Across standards and criteria for optimal sensitivity and specificity, cut offs at the PAIC15 could be 3 or 4. Guided by self report we recommend PAIC15 scores of 3 and higher to represent probable pain with sensitivity and specificity in the 0.5 to 0.7 range.

